# Quality Improvement Initiatives in Pediatric Emergency Care in Low‐Resource Settings: Addressing Patient Safety and Outcomes

**DOI:** 10.1155/ijpe/6683096

**Published:** 2026-06-26

**Authors:** Chibuike Daniel Onyejesi, Abenezer Kebede, Eslam Abady, Muhammad Azan Shahid, Chukwunonye Onyeka Ogbuji, Rayan Raed Salahaldin, Zainab Ibrahim Mohamed, Olalekan John Okesanya, Yenifer Milagros Aponte, Mohamed Alsabri

**Affiliations:** ^1^ Pediatrics, Lincoln Medical Center, Bronx, New York, USA, linmed.org; ^2^ Department of Internal Medicine, Tanta University, Tanta, Gharbia Governorate, Egypt, tanta.edu.eg; ^3^ CUNY Brooklyn College, New York City, New York, USA, cuny.edu; ^4^ Pediatrics, University of Illinois Chicago, Peoria, Illinois, USA, uic.edu; ^5^ Faculty of Medicine, Al Quds University, Jerusalem, State of Palestine, alquds.edu; ^6^ Faculty of Medicine, Suez Canal University, Ismailia, Egypt, scuegypt.edu.eg; ^7^ Neuropsychiatric Hospital Aro, Abeokuta, Nigeria; ^8^ Universidad Centroccidental Lisandro Alvarado, Barquisimeto, Lara, Venezuela, ucla.edu.ve; ^9^ St. Christopher′s Hospital for Children, Philadelphia, Pennsylvania, USA; ^10^ Althawara Modern General Hospital, Sana′a, Yemen

## Abstract

**Background:**

Pediatric emergency care (PEC) in low‐resource settings faces systemic challenges, including inadequate infrastructure, workforce shortages, and supply chain inefficiencies, which contribute to preventable morbidity and mortality. Quality improvement (QI) initiatives offer a structured framework to address these challenges, enhance patient safety, and improve clinical outcomes.

**Methods:**

A narrative review was conducted using literature identified from PubMed and Google Scholar, supplemented by reports and guidance documents from the World Health Organization and nongovernmental organizations. Search terms included combinations of “pediatric emergency care,” “quality improvement,” “patient safety,” “low‐resource settings,” “LMICs,” “triage,” “task shifting,” “telemedicine,” “digital health,” and “community health workers.” Eligible sources included primary studies, reviews, implementation reports, and policy documents addressing QI interventions, implementation barriers, facilitators, or outcomes relevant to PEC in low‐resource settings.

**Results:**

Included studies reported improvements in triage reliability, time to treatment, guideline adherence, patient‐flow organization, workforce capacity, and data availability. Several triage‐ and ETAT‐based interventions reported reductions in pediatric mortality or improved time‐sensitive care processes, whereas digital health interventions improved triage completion, referral monitoring, and real‐time decision support. Key enablers included local adaptation, staff training, audit and feedback, interdisciplinary collaboration, community engagement, and sustainable governance structures.

**Conclusion:**

Strengthening QI initiatives is essential to achieving global health goals and ensuring equitable healthcare access for children in resource‐limited settings. Sustainable, evidence‐based interventions are critical for building resilient PEC systems and optimizing workflows.

## 1. Introduction

Pediatric emergency care (PEC) in low‐ and middle‐income countries (LMICs) is constrained by unstructured triage systems, inefficient clinical workflows, and delays in life‐saving interventions. These systemic weaknesses contribute to preventable morbidity and mortality among children, particularly from conditions like malaria, pneumonia, and severe diarrheal diseases. Although these deaths are driven by multiple factors, including poverty and limited access to primary care, deficiencies in emergency care represent a critical and modifiable contributing factor [1–4].

In high‐income countries, superior outcomes in pediatric emergencies are attributable to a combination of greater financial resources, advanced infrastructure, and higher staffing levels. Within these well‐resourced systems, quality improvement (QI) initiatives have been instrumental in optimizing care delivery and patient outcomes [5]. Recognizing the importance of strengthening these services globally, the World Health Organization (WHO) has prioritized emergency and critical care as essential components of universal health coverage [6]. Although QI alone cannot close the resource gap, it is a vital tool for maximizing the effectiveness of available resources to achieve global health objectives, including the Sustainable Development Goals (SDGs) [7].

This narrative review synthesizes available evidence on QI interventions in PEC within resource‐limited settings. It focuses on implementation strategies, reported outcomes, barriers, facilitators, and lessons learned from low‐resource healthcare systems. Key thematic areas include evidence‐based triage models, workflow optimization, workforce strengthening, digital health tools, community engagement, and governance strategies.

## 2. Methods

This narrative review was conducted to synthesize evidence on QI initiatives in PEC in low‐resource settings. A literature search was performed using PubMed and Google Scholar, supplemented by reports and guidance documents from the WHO, nongovernmental organizations, and other global health implementation sources. The search focused on literature relevant to PEC, QI, patient safety, emergency triage, task shifting, digital health, community engagement, and health‐system strengthening in low‐resource settings.

The search strategy used combinations of the following keywords and medical subject headings where applicable: “pediatric emergency care,” “pediatric emergency department,” “quality improvement,” “patient safety,” “low‐resource settings,” “low‐ and middle‐income countries,” “LMICs,” “emergency triage,” “South African Triage Scale,” “Emergency Triage Assessment and Treatment,” “task shifting,” “nurse‐initiated protocols,” “telemedicine,” “digital health,” “mHealth,” “community health workers,” “implementation barriers,” and “health system strengthening.” Boolean operators were used to combine terms, including: (“pediatric emergency care” OR “pediatric emergency department”) AND (“quality improvement” OR “patient safety”) AND (“low‐resource settings” OR “LMICs”). Searches were conducted from database inception to March 2025.

Articles and reports were considered eligible if they addressed QI interventions, implementation strategies, patient‐safety initiatives, or measurable care‐process outcomes relevant to pediatric emergency or acute care in low‐resource settings. Eligible sources included primary research studies, implementation studies, observational studies, systematic reviews, scoping reviews, policy reports, and global health guidance documents. Studies were prioritized when they reported intervention details, implementation context, outcome measures, barriers, facilitators, or lessons relevant to PEC delivery.

Sources were excluded if they focused exclusively on adult emergency care, high‐income settings without relevance to low‐resource adaptation, nonemergency pediatric care, or interventions unrelated to QI, patient safety, emergency care delivery, or health‐system strengthening. Opinion pieces and narrative commentaries were not prioritized unless they provided important contextual information or implementation lessons.

Because this was designed as a narrative review rather than a systematic review or meta‐analysis, formal risk‐of‐bias scoring was not performed. However, studies were interpreted according to study design, relevance to PEC, directness of evidence, presence of measurable outcomes, and applicability to low‐resource settings. Systematic reviews and multisite studies were considered higher strength evidence, whereas single‐site observational studies, case studies, implementation reports, and expert guidance were interpreted as lower strength but useful for understanding feasibility, context, barriers, and implementation strategies.

## 3. Evidence Synthesis and Categorization of Studies

Because the included literature varied in design, scope, and methodological strength, studies were categorized according to their type of evidence and direct relevance to PEC in low‐resource settings. Primary and implementation studies were separated from systematic reviews, policy documents, and general guidance. Primary and implementation studies included original research, program evaluations, before‐and‐after studies, interrupted time‐series studies, observational studies, and implementation reports that described a QI intervention, setting, sample, outcome measure, or implementation experience. These studies are summarized in Table 1.

**Table 1:** Primary and implementation studies of quality improvement interventions relevant to pediatric emergency care in low‐resource settings.

Study	Country/setting	Study design and sample	QI intervention	Outcome measures	Main findings	Evidence strength
Molyneux et al. [8]	Malawi; pediatric outpatient/emergency unit at Queen Elizabeth Central Hospital, Blantyre	Field implementation report using routine audit data; department previously treated about 90,000 children/year, with about 12,000 admissions/year	Emergency‐care and triage training, redesigned patient flow, improved cooperation between inpatient and outpatient services, and restructuring of the physical layout of the pediatric emergency area	Inpatient mortality and patient‐flow/process improvement	Inpatient mortality reportedly decreased from 10%–18% before implementation to 6%–8% after implementation, suggesting benefit from triage, emergency‐care training, and patient‐flow redesign.	Moderate direct evidence. Highly relevant to pediatric emergency care, but uncontrolled and based on routine service data.
Dekker‐Boersema et al. [9]	Mozambique; rural district hospital emergency center	Retrospective before‐and‐after mortality analysis; 4176 pediatric admissions under 15 years	Three‐color triage system conducted by lay staff plus pediatric emergency‐care/ETAT training for clinical staff	Pediatric mortality, agreement between clinical and nonclinical triage staff, time from triage to first treatment	Pediatric mortality was 45% lower after implementation; mortality rate ratio 0.55. Agreement between clinical and nonclinical staff was 88.7%; median waiting time was 9 min for red/urgent patients, 21 min for yellow/serious patients, and 2 h and 33 min for green/least serious patients.	Moderate direct evidence. Strong pediatric emergency relevance; before–after design means residual confounding is possible.
Irimu et al. [10]	Kenya; Kenyatta National Hospital pediatric services	Uncontrolled before‐and‐after study; medical records for children with pneumonia, dehydration, and severe malnutrition; study aimed to review about 280 records per disease per year	ETAT+ training and locally adapted clinical practice guidelines for common serious childhood illnesses	Guideline adherence across 15 quality indicators; selected case‐fatality rates	Seven of 15 key quality indicators improved by more than 20 percentage points; three improved by more than 50 percentage points. Correct gentamicin prescribing for pneumonia improved from 19.9% to 88%, and pneumonia mortality decreased from 15.1% to 6.5%, although the study was not designed primarily to evaluate mortality.	Moderate direct evidence. Strongly relevant to pediatric acute/emergency care; uncontrolled design limits causal inference.
Barasa et al. [11]	Kenya; district hospitals	Cost‐effectiveness analysis using data from a cluster randomized trial; four hospitals received full ETAT+ strategy and four hospitals received partial intervention	ETAT+ strategy: clinical guidelines, training, supervision, feedback, and facilitation	Fourteen process‐of‐care indicators, cost per child admission, estimated scale‐up cost	Average quality of care was 25% higher in intervention hospitals than control hospitals. Each percentage improvement in average quality cost an additional USD 0.79 per admitted child; national scale‐up was estimated at USD 3.6 million, about 0.6% of Kenya′s annual child health budget.	Moderate‐to‐strong direct evidence. Stronger design because based on trial data, but focused mainly on process and cost‐effectiveness outcomes.
Ansermino et al. [12]	Kenya and Uganda; pediatric outpatient departments in four public tertiary facilities	Controlled interrupted time‐series implementation study; 6784 children enrolled at baseline, with 5410 at intervention sites and 6032 at control sites during implementation	Smart Triage digital platform combined with QI program, risk score, patient tracking, dashboard, weekly feedback reports, and local workflow redesign	Time to intravenous antimicrobial administration, antimicrobial use, admission rate, 7‐day mortality	Triage completion reached 98.5% at intervention sites. In Kenya, time to IV antimicrobials decreased by 98 min at the intervention site while increasing at the control site. Antimicrobial use and admission rates fell at intervention sites; 7‐day mortality decreased by 25% in Kenya and 75% in Uganda, although mortality was a secondary outcome and should be interpreted cautiously.	Moderate‐to‐strong direct evidence. Highly relevant digital QI study; interrupted time‐series design is useful, but implementation was affected by COVID‐19, drug shortages, staffing shortages, and site‐level differences.
Desmond et al. [13]	Malawi; eight primary health centers in Blantyre district	Observational implementation study; 209,174 children recorded by ETAT, with 155,931 having both mHealth and clinician triage outcome data	mHealth triage algorithm based on WHO ETAT, used by lower cadre health workers, with patient surveillance from presentation through referral and outcome	Triage concordance, referral completion, acceptability	Concordance between mHealth triage by lower cadre workers and clinician assessment was 81.6%. Of 3004 referrals, 1644 reached the tertiary site, showing feasibility but also highlighting referral‐system gaps.	Moderate direct evidence. Very relevant to low‐resource pediatric triage and referral; observational design and referral attrition limit outcome interpretation.
Wangara et al. [14]	Kenya; Kenyatta National Hospital accident and emergency department, Nairobi	Two‐part study: prospective before‐after educational intervention with 166 healthcare workers using triage vignettes, plus retrospective chart review of 2420 charts	Implementation of the South African Triage Scale with healthcare‐worker education	Triage reliability, overtriage, undertriage, sensitivity/specificity for disposition outcomes	Healthcare workers agreed with expert triage standard in 64% of scenarios and had 97% agreement allowing one‐level discrepancy. Overtriage decreased from 70.9% to 62.3% after SATS implementation; SATS sensitivity was 92.2% and specificity 37.7% for admission, death, or discharge.	Moderate supportive evidence. Relevant to emergency triage in low‐resource settings; mixed adult/pediatric emergency context, not exclusively pediatric.
Soogun et al. [15]	South Africa; urban district hospital emergency center in Durban	Retrospective chart review; 346 records reviewed	Nurse‐led South African Triage Scale audit and implementation evaluation	Time to treatment, overtriage, undertriage, documentation completeness, disposition	Average time to treatment was 59 min; only 48% of patients were treated within recommended timeframes. Overtriage was 66.7% and undertriage was 14%. However, the study excluded children under 12 years, so it should be used only as supporting evidence for SATS implementation, not as direct pediatric evidence.	Low‐to‐moderate indirect evidence. Useful for SATS implementation lessons but not direct pediatric emergency evidence because younger children were excluded.
Atuhairwe et al. [16]	Uganda; national emergency‐care telementoring program	Mixed‐methods evaluation of the first 4 months of EMS‐ECHO implementation; 2030 participants across 200 health facilities	Telementoring and remote emergency‐care learning using the ECHO model	Participation, professional mix, learner satisfaction, perceived professional isolation	The program reached 2030 health workers; doctors accounted for 37% and nurses 28% of participants. 95% rated sessions as informative, and 66% agreed or strongly agreed that sessions reduced professional isolation.	Moderate supportive evidence. Relevant to workforce capacity and emergency‐care QI, but not pediatric‐specific.
Marsh et al. [17]	Haiti; Hôpital Universitaire de Mirebalais emergency department	QI‐based ED design project using stakeholder feedback; no patient sample reported	ED redesign focused on triage area, patient flow, visibility, oxygen reliability, supply storage, infection prevention, privacy, security, and ED location within hospital	Design recommendations and operational feasibility	The study proposed seven ED design recommendations for LMICs, including dedicated triage space, visibility for ill patients, reliable oxygen systems, supply‐chain–aware storage, simple ventilation, and family/staff safety considerations.	Low‐to‐moderate supportive evidence. Useful for infrastructure and workflow QI; not an outcomes‐based pediatric study.
Ali et al. [18]	Pakistan; tuberculosis control program	Sequential explanatory mixed‐methods pilot over 3 months; 122 patient records analyzed; four field workers trained	ODK Scan paper‐to‐digital data collection system with smartphones and mobile data	Data accuracy, time for data transfer, aggregation, verification, user acceptability	ODK Scan recognized 99.2% of multiple‐choice bubble responses and 79.4% of numerical digit responses correctly. Data transfer and aggregation time decreased, but verification and form‐filling time increased; final verified ODK data accuracy was lower than manual entry in this pilot.	Low indirect evidence. Useful for data‐system QI lessons, but not pediatric emergency care and should not be presented as direct PEC evidence.

Systematic reviews, overview reviews, policy documents, and global guidance were used to support the broader narrative synthesis but were not treated as primary QI intervention studies. These secondary sources are summarized separately in Table 2. This separation was used to avoid overrepresenting review articles as original intervention evidence and to clarify the strength and directness of evidence supporting each QI strategy.

**Table 2:** Secondary evidence, guidance, and framework sources supporting the narrative synthesis.

Source	Evidence type	Focus	Population/context	Role in this review
Oliphant NP et al. [1]	Systematic review	Integrated community case management of childhood illness	Children in low‐ and middle‐income countries	Supports the role of community health workers in early illness recognition, treatment, and referral, but is used as secondary evidence rather than as a primary emergency‐department QI intervention.
Pantoja T et al. [2]	Overview of systematic reviews	Implementation strategies for health systems	Health systems in low‐income countries	Supports the discussion of implementation strategies such as training, supervision, audit and feedback, and health‐system strengthening.
Zachariasse JM et al. [19]	Systematic review and meta‐analysis	Performance of triage systems in emergency care	Emergency‐care settings	Supports the discussion of triage‐system performance, reliability, and implementation challenges, while recognizing that evidence is not limited to LMIC pediatric settings.
Burgess L et al. [20]	Systematic review	Nurse‐initiated interventions in emergency departments	Emergency‐department settings	Supports the discussion of nurse‐initiated protocols as a strategy to reduce delays and standardize early care.
Okoroafor SC and Christmals CD [21]	Scoping review	Task shifting and task sharing in Africa	African healthcare systems	Supports task shifting as a workforce strategy for resource‐limited settings, although not specific to pediatric emergency care.
Colvin CJ et al. [22]	Qualitative evidence synthesis	Barriers and facilitators to task shifting	Health‐service delivery contexts	Supports the discussion of implementation barriers and facilitators, including training, supervision, role clarity, and acceptability.
Qoseem IO et al. [23]	Review/perspective	Digital health and health equity	African healthcare systems	Supports the discussion of digital health as a tool for improving access, equity, and quality in resource‐constrained settings.
Sun L et al. [24]	Systematic review of theories, models, and frameworks	Adaptability, scalability, and sustainability of complex health interventions	Complex health interventions	Supports the sustainability and scale‐up discussion, particularly the need for adaptability and long‐term implementation planning.
Barker PM et al. [25]	Scale‐up framework	Scaling health interventions in Africa	Large‐scale improvement initiatives in Africa	Supports the discussion of leadership, local ownership, data use, adaptive implementation, and system‐level learning for sustainable QI scale‐up.

Across the primary and implementation studies, the most consistently reported improvements were in triage reliability, time to treatment, guideline adherence, patient‐flow organization, workforce capacity, and data availability. Mortality improvement was reported in some studies evaluating triage and ETAT‐based emergency‐care interventions; however, these findings should be interpreted cautiously because several studies were observational, uncontrolled, or affected by concurrent health‐system changes. The strongest direct pediatric emergency evidence came from studies evaluating ETAT/ETAT+ implementation, lay‐staff or nurse‐led triage, and digital triage platforms. Indirect studies on telementoring, emergency‐department design, and digital data systems were retained because they provide transferable implementation lessons for low‐resource settings, but they should not be interpreted as direct pediatric emergency outcome evidence.

## 4. Establishing a Strong Framework for QI in PEC

Effective QI in PEC is built on structured frameworks that refine clinical workflows and integrate data‐driven decision‐making. The foundational Model for Improvement (MFI) provides this structure, which is often executed through iterative Plan‐Do‐Study‐Act (PDSA) cycles [7]. These cycles enable the rapid testing and refinement of interventions, such as optimizing triage strategies or streamlining medication administration protocols [2]. Complementing this core model, other methodologies can be integrated to target specific issues. Lean methodology, which focuses on eliminating inefficient processes, has reported value in high‐burden, resource‐limited emergency departments [5, 17]. Although less frequently used in LMICs, Six Sigma′s emphasis on reducing care variability presents an opportunity to enhance standardization and safety in PEC [17, 26, 27]. The strength of evidence for these QI methods is uneven. General QI frameworks and health‐system implementation strategies are supported by reviews and evidence‐based guidance [2, 3], whereas several pediatric emergency examples from LMICs rely on observational studies, implementation reports, or single‐site experiences, which limits causal interpretation [17, 14, 15].

Successful QI implementation depends on key enabling factors. Interinstitutional collaboration, for example, allows providers to share solutions for large‐scale challenges, such as reducing sepsis mortality in PEC [1, 28]. Equally critical is the local adaptation of QI frameworks. Efforts in Haiti demonstrated how designing adaptable triage areas and scalable staffing plans tailored to local resource constraints can improve outcomes, particularly by improving patient flow, operational organization, and the functionality of emergency services [17].

However, implementing these frameworks in resource‐constrained environments faces considerable barriers. These include a limited availability of personnel trained in QI, significant financial and time constraints [2, 28], and unreliable data systems that hinder progress tracking [4, 5]. Cultural and systemic issues, such as resistance to change or hierarchical healthcare structures, can further complicate implementation efforts [4, 5]. Beyond training, successful implementation is consistently facilitated by fostering a nonpunitive culture for error reporting, securing visible commitment from local hospital leadership, and utilizing low‐cost, paper‐based data tracking tools where electronic systems are unreliable. Conversely, major barriers include high staff turnover that disrupts PDSA cycles, fragmented supply chains that prevent the standardization of interventions, and the cognitive burden on overworked staff asked to adopt new protocols without additional resources. Addressing these unique challenges is essential for enhancing PEC and ensuring equitable access to high‐quality healthcare [17, 27].

## 5. Strengthening Emergency Department Efficiency: Optimized Triage and Fast‐Track Protocols

Overcrowding in PEC remains a significant challenge, particularly in LMICs where patient loads frequently exceed system capacity. Effective triage and fast‐track protocols are critical in improving patient flow, prioritizing critical cases, and reducing emergency department congestion [19, 29].

Standardized triage models—such as the Australasian Triage Scale, the Canadian Triage and Acuity Scale, the Emergency Severity Index, and the Manchester Triage System—enhance clinical decision‐making and reduce treatment delays [14]. However, implementation gaps in LMICs, including insufficient staff training and adherence challenges, necessitate local adaptations of these models [30]. Fast‐track pathways for noncritical cases have been shown to optimize emergency department efficiency and reduce wait times, as demonstrated in various settings.

### 5.1. Illustrative Example: Adaptations and Outcomes of the South African Triage Scale (SATS) in Low‐Resource Settings

The SATS, developed specifically for resource‐limited settings in South Africa, has demonstrated strong reliability and validity across various studies conducted in similar environments [14]. It is designed to be straightforward, quick to use, and beneficial for nursing assistants in the emergency department [15].

Its effective implementation involves adapting it to handle fluctuating patient volumes and aligning its protocols with established healthcare practices, ensuring staff acceptance and optimizing triage processes for greater efficiency in resource‐constrained emergency settings [14].

An evaluation of SATS in a busy urban district hospital in Durban, South Africa, shed light on its practical application and outcomes:

• Positive impact: A review of 346 patient records indicated that SATS helped streamline triage processes, confirming that nurse‐led triage using SATS can be effective in low‐resource settings [15].
• Identified shortcomings: Despite its benefits, the study highlighted areas for improvement, including an increased average time to treatment, with just 48% of patients receiving care within recommended time limits. Essential bedside tests were often omitted, and documentation was frequently incomplete [15].

This evaluation illustrates that while SATS is a valuable tool, its optimal effectiveness relies on ongoing training, robust adherence, and better resource allocation to truly enhance patient care and operational efficiency in emergency departments [15].

Additional primary studies support the importance of triage‐focused QI interventions in PEC in low‐resource settings. In Malawi, implementation of improved triage, emergency‐care training, patient‐flow redesign, and restructuring of the pediatric emergency area was associated with reduced inpatient mortality among children [8]. Similarly, in rural Mozambique, a three‐color triage system conducted by lay staff, combined with Emergency Triage, Assessment, and Treatment training for clinical staff, was associated with reduced pediatric mortality and shorter time from triage to treatment for urgent cases [9]. These findings suggest that locally adapted triage systems can improve emergency‐care prioritization and patient flow, although most available studies remain observational or before‐and‐after in design.

## 6. Expanding the Role of Nurses and Task Shifting as QI Strategies for Enhanced PEC

Addressing healthcare workforce shortages in PEC in LMICs is a critical QI imperative. QI initiatives focusing on workforce optimization, such as empowering nurses with expanded protocols and implementing structured task shifting, are essential for mitigating these shortages, reducing care delays, and improving patient outcomes [20].

Nurse‐initiated treatment protocols serve as a direct QI intervention to standardize and expedite care. Examples in pediatric pain management and respiratory distress interventions have demonstrated measurable improvements in time‐to‐treatment and overall patient comfort. The expedited administration of analgesia and early respiratory support, through standardized nurse‐led interventions, has been linked to improved recovery rates [31, 32]. Successful and consistent protocol adherence, a key QI metric, necessitates structured training programs and ongoing provider support [20, 33].

Task shifting, defined as the strategic delegation of clinical responsibilities to nonspecialist healthcare workers following targeted training, is another effective QI strategy. This approach has proven instrumental in reducing pediatric emergency department bottlenecks and improving access to care [21, 34]. Kenya′s Task Sharing Policy (TSP), for instance, exemplifies a successful QI model that utilized task delegation to extend emergency care coverage without compromising patient safety [22, 35]. Despite inherent logistical and legal challenges, task shifting remains an invaluable QI solution for addressing workforce shortages and improving the quality of service delivery in resource‐limited emergency settings [36].

Training‐based QI interventions also show measurable benefits in pediatric acute care. In Kenya, implementation of ETAT+ training and locally adapted clinical practice guidelines improved several quality‐of‐care indicators for seriously ill children, including assessment, classification, treatment, and follow‐up care during the first 48 h of admission [10]. A related Kenyan evaluation of a multifaceted ETAT+ strategy, including guidelines, training, supervision, feedback, and facilitation, found improvement in pediatric quality‐of‐care indicators and suggested that such interventions may be cost‐effective when scaled through district hospitals [11]. These studies highlight that training alone is insufficient unless paired with supervision, feedback, and locally adapted clinical guidelines.

## 7. Leveraging Telemedicine and Digital Health as QI Enablers in PEC

Telemedicine and digital health are powerful QI enablers, leveraging technology to address geographical barriers, enhance clinical decision‐making, and improve provider training in PEC. These innovations facilitate remote specialist consultations, expand educational opportunities, and support real‐time QI decision‐making by providing critical data [23].

Initiatives like the Extension for Community Healthcare Outcomes (ECHO) program demonstrate how telemedicine functions as a QI platform for continuous professional development. ECHO enhances provider‐to‐provider education, ensuring healthcare workers in underserved regions receive expert clinical guidance that directly translates to improved quality of care [37]. An EMS‐ECHO pilot program implemented in Uganda, for example, successfully engaged a diverse range of healthcare professionals (including prehospital teams, doctors, and nurses) and stakeholders, attracting over 2000 participants in just 4 months. This broad engagement facilitated knowledge transfer and fostered a comprehensive approach to addressing quality gaps in emergency care delivery and resource allocation [16].

Recent pediatric digital‐health studies provide more direct evidence for QI in low‐resource emergency and acute‐care settings. In Kenya and Uganda, Smart Triage combined a digital risk score, patient tracking, dashboard feedback, and local QI workflows to improve prioritization of acutely ill children presenting to health facilities [12]. In Malawi, the ASPIRE mobile health (mHealth) primary ETAT package used a mobile triage algorithm to support lower cadre health workers in identifying and referring children with severe illness [13]. These studies demonstrate the potential of digital tools to strengthen triage, referral pathways, and real‐time monitoring, but they also show that technology must be supported by staff training, workflow redesign, referral capacity, and data‐verification systems.

The adoption of mobile triage applications and digital decision‐support tools directly contributes to QI by reducing emergency department congestion, improving the efficiency of patient flow, and enhancing pediatric patient outcomes [38–40]. Furthermore, advanced digital solutions such as wearable biosensors, remote patient monitoring, and AI‐driven diagnostic algorithms are reshaping PEC delivery, especially in rural LMICs where traditional healthcare infrastructure is often inadequate [41]. By integrating these tools, healthcare systems can implement data‐driven QI initiatives to extend reach, reduce disparities, and continuously improve patient safety in resource‐constrained environments [16, 42].

## 8. Community and Parental Engagement: A Cornerstone for Pediatric Emergency Preparedness

Community and parental engagement is essential in reducing pediatric emergency response delays, improving health‐seeking behavior, and ensuring timely intervention. Large‐scale community‐based training programs have demonstrated success in enhancing pediatric emergency preparedness and reducing preventable mortality [43].

Parents and community members are often the first to recognize signs of distress in a child; therefore, their knowledge and immediate actions are crucial to timely and effective pediatric emergency response. However, there are gaps in information about when to seek treatment and what qualifies as an emergency in many low‐resource situations [43, 44]. Many parents and caregivers may be unaware of signs that call for emergency medical attention. This knowledge gap can delay appropriate care and increase the risk of poor outcomes [44].

Several QI initiatives have attempted to bridge these knowledge gaps by leveraging community and parental engagement to improve PEC outcomes.

The Integrated Community Case Management (iCCM) initiative in Malawi and Uganda has effectively trained community health workers to recognize early signs of pediatric illness, facilitate urgent referrals, and administer immediate care interventions [44, 45]. Similarly, Bangladesh′s Rural Advancement Committee (BRAC) has implemented culturally tailored public health education programs that improve parental recognition of pediatric emergency symptoms [46].

Localized health messaging, adapted to cultural and linguistic contexts, plays a critical role in ensuring community engagement. Addressing common misconceptions surrounding emergency care—while leveraging trusted community figures to disseminate information—has significantly improved early treatment‐seeking behavior [47, 48]. These strategies underscore the necessity of integrating community‐based education and parental involvement into PEC frameworks [49, 50].

## 9. Strengthening Community Engagement: Culturally Tailored Public Health Messaging

Culturally tailored public health messaging is essential for enhancing community engagement and improving PEC outcomes in low‐resource settings. Effective communication strategies must align with local beliefs, customs, and languages to ensure accessibility and acceptance of medical interventions. Traditional health perceptions often lead to delays in seeking emergency care [47]. For example, febrile illnesses may be attributed to supernatural causes in some communities, delaying treatment. Healthcare organizations can address these barriers by developing culturally relevant educational campaigns that reinforce evidence‐based medical care while respecting local traditions [48].

Successful initiatives in South Asia and Sub‐Saharan Africa demonstrate that incorporating local languages, culturally relevant analogies, and trusted community leaders significantly improves awareness and early intervention rates [49, 50]. Public health messaging that uses relatable symbols, metaphors, and traditional leaders to communicate pediatric emergency symptoms fosters timely medical intervention and reduces healthcare hesitancy [46, 50]. Sustainable interventions require continuous investment in culturally informed education strategies to build trust and improve emergency response.

## 10. Data Collection, Monitoring, and Evaluation: Advancing Evidence‐Based PEC

Data collection, monitoring, and evaluation are fundamental to improving PEC quality and patient safety. However, the absence of electronic health records (EHRs) and reliance on fragmented manual documentation hinder efficient decision‐making in many low‐resource settings [51, 52]. Additionally, poor internet connectivity, lack of personnel training, and data security concerns impede accurate data analysis [53, 54].

Innovative digital health solutions have shown promise in overcoming these challenges. In Pakistan, the Open Data Kit (ODK) Scan system was piloted in a tuberculosis control program to digitize paper‐based records. The system correctly recognized 99.2% of multiple‐choice bubble responses and 79.4% of numerical digit responses, and it reduced data transfer and aggregation time; however, verified ODK data accuracy was lower than manual data entry, highlighting the need for workflow adaptation, user training, and data‐verification processes when introducing digital tools in low‐resource settings [18]. In Malawi and Rwanda, mHealth applications have improved disease surveillance, real‐time data entry, and emergency response coordination [55, 56].

To bridge the data gap in pediatric emergency departments, healthcare systems must address “digital determinants of health,” including affordability, digital literacy, and accessibility. Developing low‐bandwidth EHR systems, designing mobile‐compatible data collection tools, and training healthcare providers in digital solutions are critical for ensuring equitable access to quality data [57, 58].

## 11. Culturally Responsive PEC: Integrating Local Beliefs and Practices

Culturally adapted care models enhance accessibility and acceptance of pediatric emergency interventions. Healthcare systems must integrate local languages, beliefs, and practices into service delivery to foster trust and ensure adherence to medical recommendations [59]. Collaborating with community leaders and leveraging traditional communication methods improves the effectiveness of culturally sensitive public health campaigns [60, 61].

In Latin America and Africa, culturally tailored educational programs addressing financial concerns and misconceptions about modern medicine have improved parental understanding of emergency symptoms, leading to timely medical intervention [62]. Integrating QI methodologies into culturally adapted interventions ensures sustained impact and long‐term healthcare resilience.

## 12. Policy and Governance: Building Support for QI

Government policies and regulatory frameworks play a critical role in advancing QI in PEC. Programs such as the Affordable Care Act (ACA) and the Children′s Health Insurance Program Reauthorization Act (CHIPRA) have improved pediatric healthcare through standardized quality measures and research‐driven funding [63, 64].

Overcoming regulatory barriers and building partnerships with NGOs and international organizations are crucial for improving healthcare delivery and scaling QI programs in LMICs. Regulatory challenges, such as restrictive licensing, importation issues for medical supplies, and limited scope of practice for healthcare professionals, can impede access to timely PEC [65]. To address these barriers, regulations should be streamlined, approval processes for critical supplies expedited, and healthcare workers supported with legal and professional assistance. NGOs play a key role by leveraging local expertise to navigate these challenges. International organizations like the WHO and UNICEF contribute through technical assistance, policy advocacy, and funding for essential programs [65]. These partnerships help align local health systems with global standards and improve access to vaccines, medicines, and training. By collaborating, local governments, NGOs, and international organizations can overcome regulatory barriers, create sustainable healthcare solutions, and enhance the quality of emergency care for children [64].

## 13. Scaling and Sustaining QI Initiatives in PEC

Ensuring the long‐term sustainability and scalability of QI initiatives in PEC within LMICs demands a multifaceted approach. This requires initial adaptability to unique local challenges, supported by stable financing, ongoing workforce training, and collaborative community engagement [24, 66].

Effective scaling involves integrating principles for sustained growth into the QI process from its inception. This means not merely implementing an intervention, but systematically developing it for broader application and long‐term viability. Customizing interventions to local contexts remains paramount, as highlighted by studies such as Lagos State′s Nigeria Healthcare Quality Initiative (NHQI), where implementation designs were tailored to diverse facility types, governance structures, and resource levels despite a shared theory of change [66]. Addressing persistent challenges like staff turnover, infrastructure limitations, and varying leadership commitment requires flexible, adaptive implementation strategies aligned with local healthcare priorities.

Barker et al. [25] emphasize that successful scale‐up of health interventions requires more than replication of a fixed model; it depends on leadership, system‐level learning, local ownership, data use, and adaptation to context, all of which are essential for sustaining QI initiatives in PEC in low‐resource settings.

To achieve successful and sustainable scale‐up, specific barriers must be addressed through integrated QI efforts:

1. Leadership and governance: Robust governance frameworks are essential to sustain QI efforts. In Lagos, public hospitals benefited from structured oversight, whereas private hospitals faced challenges due to a lack of unified coordination. Empowering local leaders and establishing clear governance provides the necessary environment for QI to thrive and expand [66].
2. Capacity building: High staff turnover necessitates continuous investment in workforce skills. QI initiatives must build in long‐term capacity‐building models, including mentorship programs and ongoing education to ensure sustained competencies and prevent knowledge loss [66].
3. Financial sustainability: Strategic planning for financial resources is key. Prioritizing cost‐effective, scalable interventions and blending funding sources—including government, NGOs, and international donors—can ensure the longevity and growth of QI efforts [66].
4. Data‐driven decision‐making: Robust monitoring and evaluation systems are fundamental for both initial implementation and scaling. Strengthening health information systems and standardizing data collection allow for real‐time tracking, inform necessary adaptations, and demonstrate impact, as evidenced by disparities in accountability between public hospitals and primary health centers in Lagos [25, 66].

Sharing best practices via regional QI networks and collaboratives accelerates the adoption of successful interventions and fosters peer‐to‐peer learning. Furthermore, leveraging low‐cost, high‐impact interventions—such as task shifting and mHealth technology, as discussed earlier in this paper—directly addresses infrastructure limitations and ensures QI efforts remain sustainable and widely applicable. By proactively integrating these strategic approaches within the QI framework, initiatives in pediatric emergency departments can be successfully scaled and sustained in LMICs, ultimately improving healthcare quality and patient outcomes. These recommendations are summarized in Table 3.

**Table 3:** Key recommendations for scaling and sustaining quality improvement (QI) initiatives in low‐resource settings.

Recommendation	Summary of keypoints
Leadership and governance	Establish clear governance frameworks and empower local leaders. Utilize structured oversight mechanisms suitable for both public and private health facilities.
Capacity building	Implement continuous education, mentorship programs, and long‐term models to address staff turnover and sustain competencies in QI.
Financial sustainability	Blend funding sources (government, NGOs, and donors). Prioritize cost‐effective, high‐impact interventions that require minimal additional resources for easier scaling.
Data‐driven decision‐making	Strengthen health information systems through simplification and standardization of data collection. This enables real‐time tracking, informed decision‐making, and demonstration of impact for successful scale‐up.

## 14. Conclusion and Call to Action

Implementing sustainable QI interventions in PEC is vital for reducing child mortality and enhancing healthcare system efficiency. Continued investment in evidence‐based practices, culturally responsive interventions, and collaborative partnerships will drive transformative change in low‐resource settings. Through structured QI initiatives, pediatric emergency departments can build resilient, patient‐centered healthcare systems that improve outcomes and advance global health equity.

**Nomenclature**
ACA: Affordable Care Act
BRAC: Bangladesh Rural Advancement Committee
CHIPRA: Children′s Health Insurance Program Reauthorization Act
EHRs: electronic health records
ETAT: Emergency Triage, Assessment, and Treatment
LMICs: low‐ and middle‐income countries
mHealth: mobile health
NGO: nongovernmental organization
ODK: Open Data Kit
PEDs: pediatric emergency departments
QI: quality improvement
SATS: South African Triage Scale
SDGs: Sustainable Development Goals
WHO: World Health Organization

## Author Contributions

C.D.O. is considered the first author. He participated in the drafting of the article and reviewed and revised the manuscript, applying reviewers′ corrections. A.K. and E.A. are considered second authors; they contributed to the conception, formulation, drafting of the article, and reviewed and revised the manuscript. M.A.S., C.O.O., R.R.S., Z.I.M., O.J.O., Y.M.A., and M.A. participated in writing and revising the final manuscript.

## Funding

No funding was received for this manuscript.

## Disclosure

All authors approved the final manuscript as submitted.

## Ethics Statement

The authors have nothing to report.

## Conflicts of Interest

The authors declare no conflicts of interest.

## Data Availability

Data sharing is not applicable to this article as no datasets were generated or analyzed during the current study.
